# Supplementary material for: mirror determines the far posterior domain in butterfly wings
Source: eLife. 2025 Jun 25;13:RP96904. doi: 10.7554/eLife.96904 (PMC12194122; doi:10.7554/eLife.96904)
Supplement: Supplementary file 1. — A maximum likelihood phylogeny of J. coenia, H. erato lativitta, T. castaneum, A. mellifera, and D. melanogaster Iroquois Complex genes confirms that JC_02269-RA is the ortholog of mirror. [file elife-96904-supp1.docx]

Supplementary File 1.

**
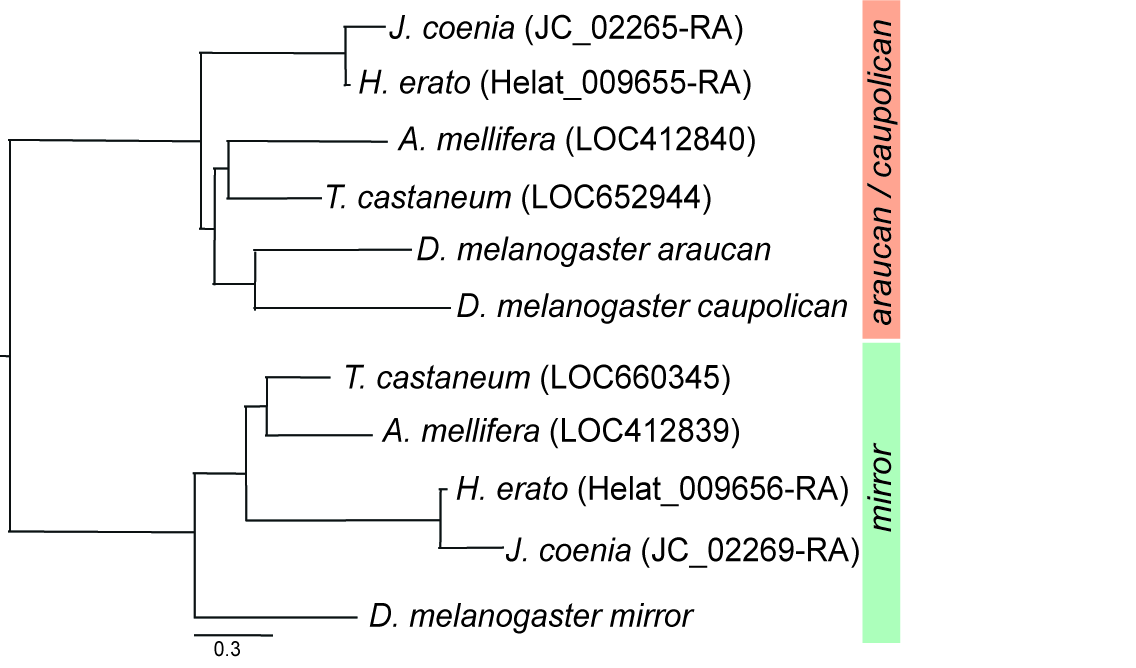
**

**Identification of *mirror* ortholog in *J. coenia*.** A maximum likelihood phylogeny of *J. coenia*, *H. erato lativitta*, *Tribolium castaneum*, *Apis mellifera*, and *D. melanogaster Iroquois* *Complex* genes confirms that JC_02269-RA is the ortholog of *mirror*.
