# Supplementary material for: mirror determines the far posterior domain in butterfly wings
Source: eLife. 2025 Jun 25;13:RP96904. doi: 10.7554/eLife.96904 (PMC12194122; doi:10.7554/eLife.96904)
Supplement: Supplementary file 2. [file elife-96904-supp2.docx]

Supplementary File 2.

| **sgRNA concentration (ng/μl)** | **Eggs  Injected** | **Hatched** | **Adults  Emerged** | **Mutants** |
| --- | --- | --- | --- | --- |
| 487 | 142 | 25 | 4 | 2 |
| 391 | 96 | 33 | 6 | 3 |
| 335 | 163 | 32 | 15 | 4 |
| 281 | 98 | 11 | 1 | 0 |
| 175 | 149 | 52 | 19 | 8 |
| 125 | 252 | 79 | 24 | 9 |
| 88.5 | 142 | 41 | 30 | 3 |
| **Total** | **1042** | **273** | **99** | **29** |
