# Supplementary material for: mirror determines the far posterior domain in butterfly wings
Source: eLife. 2025 Jun 25;13:RP96904. doi: 10.7554/eLife.96904 (PMC12194122; doi:10.7554/eLife.96904)
Supplement: Supplementary file 3. — Sanger sequencing results from three different mirror mutant individuals. Each line denotes a 394 different wild-type (WT) or mutant allele with indels indicated in red and the PAM site in green. [file elife-96904-supp3.docx]

Supplementary File 3.

**
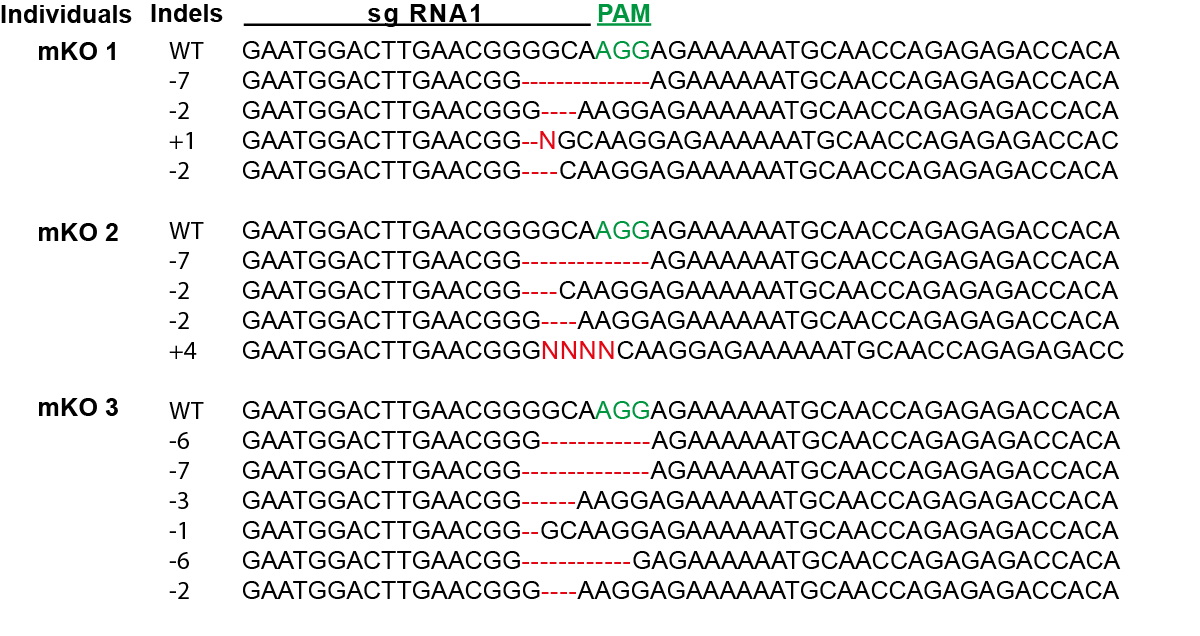
**
**Supplementary File 3. Sanger sequencing confirms mutations at CRISPR sgRNA site in *mirror* mKOs.** Sanger sequencing results from three different *mirror* mutant individuals. Each line denotes a different wild-type (WT) or mutant allele with indels indicated in red, and the PAM site in green.
